# Visualization Study of Oil-in-Water-in-Oil (O/W/O) Double Emulsion Formation in a Simple and Robust Co-Flowing Microfluidic Device

**DOI:** 10.3390/mi8090268

**Published:** 2017-09-01

**Authors:** Pengfei Lu, Liangyu Wu, Xiangdong Liu

**Affiliations:** 1 School of Hydraulic, Energy and Power Engineering, Yangzhou University, Yangzhou 225127, China; pfabc0826@126.com (P.L.); lywu@yzu.edu.cn (L.W.); 2 School of Energy and Environment, Southeast University, Nanjing 210096, China

**Keywords:** visualization, co-flowing, double emulsion, microfluidic

## Abstract

A simple and robust co-flowing microfluidic device for double emulsion preparation is designed and assembled to visually study the double emulsion formation by the use of a microscope and high-speed camera. Using a visualization system, the transient processes of double emulsion formation in co-flowing stream are observed and recorded for a variety of flow rates. The effects of flow rates of each fluid on flow modes, drop sizes, and polydispersities are examined and analyzed. The results indicate that the detaching of the inner drops accelerates the detaching of the outer drops and speeds up the drop formation process of double emulsions. The manipulation of flow rates is capable to actively control the sizes of the inner and outer drops as well as the number of inner drops encapsulated. Without surface modification, the microfluidic device produces a variety of emulsions, including the single-core and multi-core O/W/O double emulsions as well as binary emulsions of single and double emulsions. The proposed co-flowing microfluidic device is highly desirable in producing double emulsions in an easy and cheap way.

## 1. Introduction

Monodispersed drops in emulsions [1,2] are ideal vessels for reactions in small scale [3], control release [4] and drug delivery [5]. Additional protection of the inner drops can be achieved by using double emulsions, especially when the inner drops contain active substances [6]. Attributed to the unique and adjustable core–shell structure, double emulsions represent a developing trend in the preparation of various functional materials [7] and biochemical analysis [8]. Traditional methods to produce double emulsions usually involve rough oscillations which may cause uncontrollability in drop size and monodispersity [9]. With advantageous control over both local and global flow fields, producing double emulsions through microfluidic approaches has become one of the most promising techniques [10].

Among various microfluidic devices, co-flowing configuration is the most commonly used design with all fluids flowing coaxially [11]. The emulsion drops are formed under the cooperation of viscous force and interfacial tension [12,13]. Generally, the fluid that has been dispersed is entirely embraced by the fluid flowing continuously in co-flowing streams which makes surface treatment unnecessary. Cramer et al. [14] investigated the drop formation of aqueous solutions in fully developed co-flowing streams of sunflower oil in a wide range of flow conditions. The relevant parameters that determine the drop breakup modes and the drop sizes are discussed comprehensively. Utada et al. [12] studied the dominant force during the formation of drops in co-flowing streams and distinguished two classes of transition from dripping to jetting. The combination of two co-flowing configurations allows the production of double emulsion drops. Oh et al. [15] molded a microfluidic device that produce double emulsions in a two-step approach. The inner drops were produced at the tip of the first pipette, carried downstream, and encapsulated by the middle fluid at the tip of the second pipette. Integrated with immediate photopolymerization, the shells of double emulsions were polymerized, forming polymer microcapsules in situ. Additional scalability and flexibility was achieved by Wang et al. [16] who developed a modular microfluidic device comprised of a drop maker, connector, and liquid extractor. Using these modules, the multi-core, multi-component double emulsions and high-order emulsions in a variety of forms can be produced by controlling the flow rates.

The formation processes of double emulsions involve two nesting interfaces that interact with each other, leading to complicated mechanisms that still lack comprehensive experimental insights. The design and optimization of microfluidic devices require full exploration of the dynamic behaviors of the emulsification processes. Therefore, a three-dimensional co-flowing microfluidic device is designed and assembled in the current investigation with the outlets of the inner fluid and middle fluid on the same cross section where the double emulsions are formed in one step. Based on this microfluidic device, the hydrodynamic behaviors of various drop formation modes are examined via a visualization system using a microscope and high-speed camera. The influence of flow rates on flow modes, drop sizes, and polydispersities are examined and analyzed.

## 2. Materials and Methods 

### 2.1. Fabrication of the Microfluidic Device

**Figure 1:**
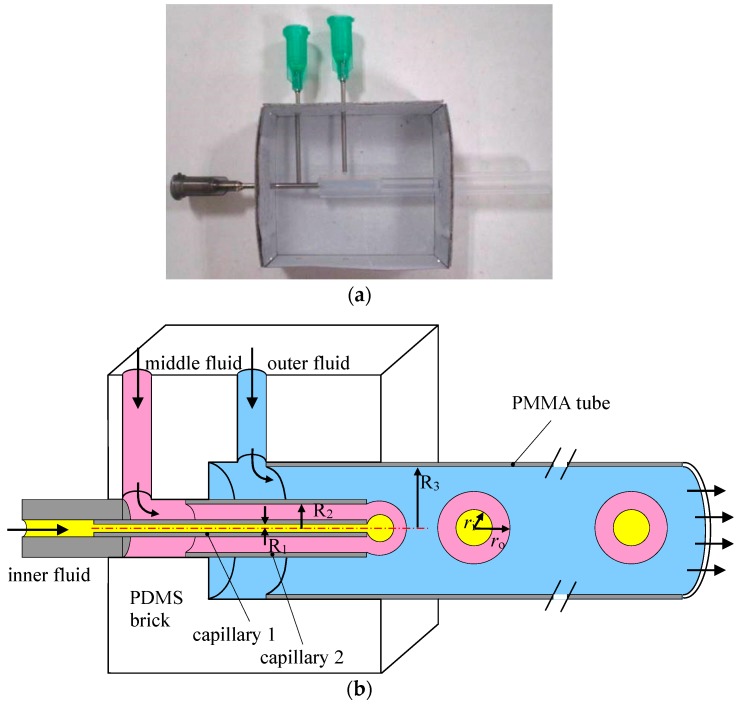
(**a**) Fabrication of the PDMS brick; (**b**) Schematic illustration of the co-flowing microfluidic device.

A simple and robust co-flowing microfluidic device for the production of O/W/O double emulsion is assembled at low cost without requirement for the modification of surface wettability. First, a poly(dimethylsiloxane) (PDMS) brick with fluid passages is molded as shown in Figure 1a. Nested needles and PTFE tubes are mounted at a paper box then liquid PDMS premixed curing agent is poured into the box. After curing, the needles and PTFE tubes are removed, leaving a PDMS brick with a stepped through-hole and two side holes. Since the through-hole is aligned when fabricating, the steel capillaries are centered after assembled into the PDMS brick. A solid rod is borne against the exit of Capillary 2 after it has been inserted from the right side. Then capillary 1 is inserted from the left side until it cannot be pushed any further. Hereby, the exits of the two capillaries are made at the same cross section. The PDMS brick is used for the transportation of the fluids and alignment of the capillaries and tubes as shown in Figure 1b. Steel capillaries pulled off from 16 gauge and 23 gauge needles are then inserted into the PDMS brick to form co-flowing passages for the inner and middle fluids. Capillary 1 (OD 0.62 mm, ID 0.34 mm) is nested at the end of a PTFE tube that insert into the inlet passage of the PDMS brick serving as the inlet for the inner fluid. While capillary 2 (OD 1.6 mm, ID 1.32 mm) is inserted at the center of the PDMS brick serving as the inlet of the middle fluid that surrounding Capillary 1 forming co-flowing stream. A transparent polymethyl methacrylate (PMMA) tube (OD 6 mm, ID 4 mm) is inserted at the outlet passage of the PDMS brick to form co-flowing stream of the outer fluid surrounding the middle and inner fluid as well as collect the generated emulsions. Each fluid is pumped by a separate syringe pump that is connected by PTFE tubes to the PDMS brick. The exits of the two steel capillaries are adjusted to be at the same cross section so that the formation of double emulsion is accomplished in a single step. Since the inner fluid is completely embraced by the middle fluid, no special surface treatment is needed.

### 2.2. Materials

The PDMS brick is made of SYGRID 184 (Dow Corning Co., Shanghai, China) and cured at 85 °C for 4 h. Silicone oil (Dow Corning Co., Shanghai, China) with the viscosity of 20 mPa·s and 500 mPa·s is used as the inner fluid and outer fluid, respectively, while 5% (*w*/*w*) solution of Poly(vinyl alcohol) 1788 (Aladdin Biochemical Technology Co., Shanghai, China) was used as the middle fluid. Three syringe pumps (LSP02-1B, Longer Precision Pump Co., Baoding, China) are used to inject the fluids into the devices. The visualization system is comprised of a microscope (SZX7, Olympus, Tokyo, Japan), a high-speed camera (500K-M2, Photron, Tokyo, Japan), a computer, and a light source. All experiments are conducted at constant room temperature of 22 °C.

## 3. Results and Discussion

To quantitatively describe the fluid dynamics during the formation of double emulsions, the distances from the front of the inner and outer interfaces to the outlets of the inner and middle fluid are measured as the lengths of the inner and outer drop (denoted as *l*_i_ and *l*_o_ in Figure 1) and nondimensionalized by the inner radius of the collecting PMMA tube *R*_3_ as *L*_i_ = *l*_i_/*R*_3_ and *L*_o_ = *l*_o_/*R*_3_. Non-dimensional radii of the inner drop *R*_i_ = *r*_i_/*R*_3_ and outer drop *R*_o_ = *r*_o_/*R*_3_ are also measured to evaluate the dependence of drop size and size distribution on flow rates. The non-dimensional flow time is defined as *τ* = *t*/(*R*_1_/*v*_i_) where *v*_i_ = *q*_i_/(π·*R*_1_^2^) is the mean velocity of the inner fluid and *q*_i_ is the flow rate of the inner fluid.

### 3.1. Continuous Production of Single-Core Double Emulsions

**Figure 2:**
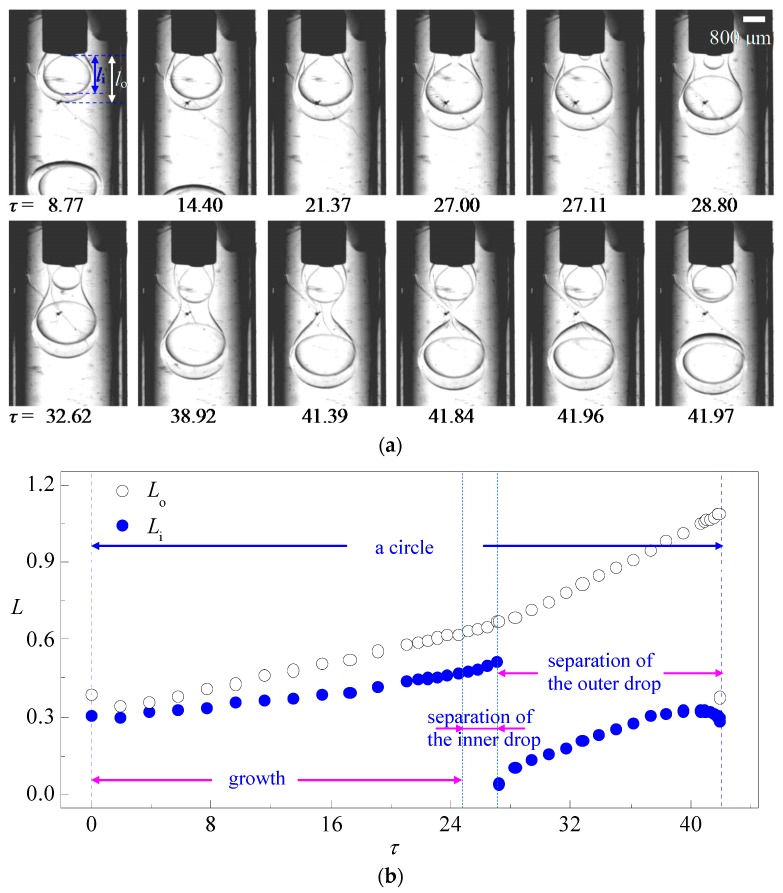
A typical case of single-core double emulsion formation: (**a**) time sequence of interface morphologies; (**b**) variation of the length of the inner and outer drop in one drop formation circle (*q*_i_ = 2.5 mL/h, *q*_m_ = 2.5 mL/h, *q*_o_ = 30 mL/h).

As demonstrated in Figure 2, the formation of a single-core double emulsion drop can be divided into three stages: growth, separation of the inner drop, and separation of the outer drop. During the growth stage, the three fluids form two almost spherical interfaces that attach to the capillary tips indicating the leading role of interfacial tension. Both drop lengths *L*_i_ and *L*_o_ increase with continuous injection of the fluids while *L*_o_ increases slightly faster. The slight decrement of *L*_i_ and *L*_o_ at first is caused by the retraction of the outer interface after the detachment of the last double emulsion drop. When the inner drop reaches a critical volume, the separation of the inner drop begins with a visible neck connect the inner drop with the tip of the inner capillary. Once the neck is formed, it shrinks rapidly under the effect of interfacial tension and eventually pinches off in a short time as shown in Figure 2b. After that, a sphere inner drop is formed inside the outer drop. The viscous force from the middle fluid carries the inner drop downstream inside the outer drop and a neck is formed at the outer interface downstream of the inner interface (Figure 2a, *τ* = 28.8), indicating the separation of the outer drop. As plotted in Figure 2b, *L*_o_ turns up a discontinuous faster increment after the separation of the inner drop, attributed to enhancement of middle fluid flow caused by the detaching of the inner drop. Likewise, separation of the outer drop is also characterized by the shrinking of the neck and pinching off of the interface. Note that the outer interface squeezes the middle fluid upstream during pinching-off leading to the front of the inner interface been pushed backwards as shown in Figure 2b. These interactions between the interfaces contribute to the radial growth of both inner and outer drops. After the detachment of the outer drop, sharp interfaces (Figure 2a, *τ* = 41.96–41.97) are retracted by the interfacial tension, lengths of both inner and outer drop decrease in a short period of time.

### 3.2. Continuous Production of Multi-Core Double Emulsions

**Figure 3:**
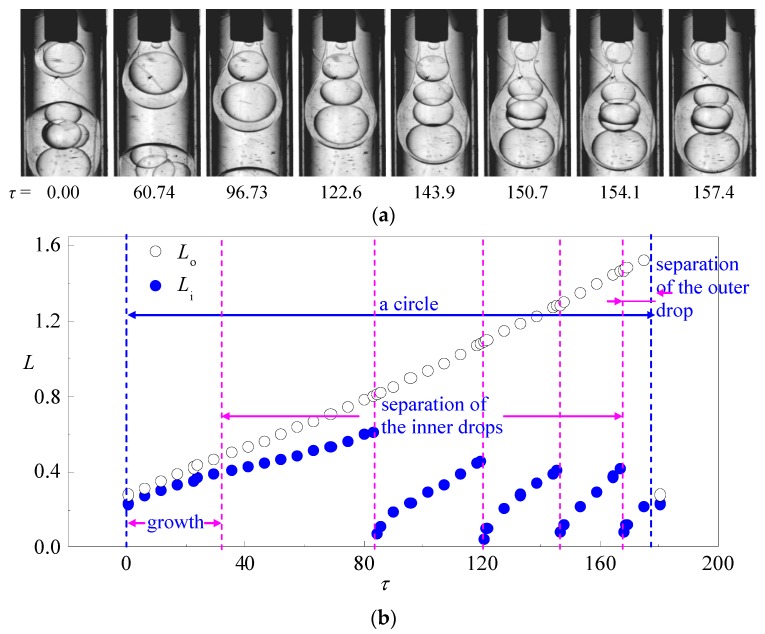
A typical case of single-core double emulsion formation: (**a**) time sequence of interface morphologies; (**b**) variation of the length of the inner and outer drop in one drop formation circle (*q*_i_ = 2.5 mL/h, *q*_m_ = 2.5 mL/h, *q*_o_ = 5 mL/h).

Under the condition of low flow rate of the outer fluid, double emulsions with multi-cores (Figure 3) can be produced by the co-flowing microfludic device. The variation of drop lengths *L*_o_ and *L*_i_ indicate that the formation process of multi-core double emulsion can be divided into several stages: growth, separation of the first inner droplet, separation of the second inner droplet, separation of the *N*th inner droplet, and separation of the outer drop. The growth stage is similar as that during the formation of double emulsions with single core. However, due to the insufficient viscous force from the outer fluid which cannot overcome the interfacial tension of the outer interface, the outer drop continues growing after the detaching of the first, second, and (*N* − 1)th inner drops. As plotted in Figure 3b, except the first inner drop, the subsequent inner drops present similar growth and separation features. However, the detached inner drops still remain inside the outer interface leading to pressure increment inside the outer interface that each inner drop is formed under higher resistance than its previous one. Hence, the growing time of each inner drop is shorter than the previous one which leads to a comparatively smaller drop size compared to its previous inner drop. Since the growing and detaching processes of the inner drop are confined by the outer interface in our device, the afore detached inner drops accumulate inside the outer interface and squeeze each other including the growing interface at the tip of the inner capillary. As a result, the inner drops in the multi-core double emulsions generated by our device are polydispersed. Note that monodispersed multi-core double emulsions can be produced by two-step co-flowing microfluidic devices in which the inner drops are generated upstream by the first order co-flowing junction before entering the outer interface at the second co-flowing junction [17]. However, surface wettability modification is required [18] in the two-step devices to guarantee the correct fluid to be emulsified.

### 3.3. Alternative Production of Single and Double Emulsion

**Figure 4:**
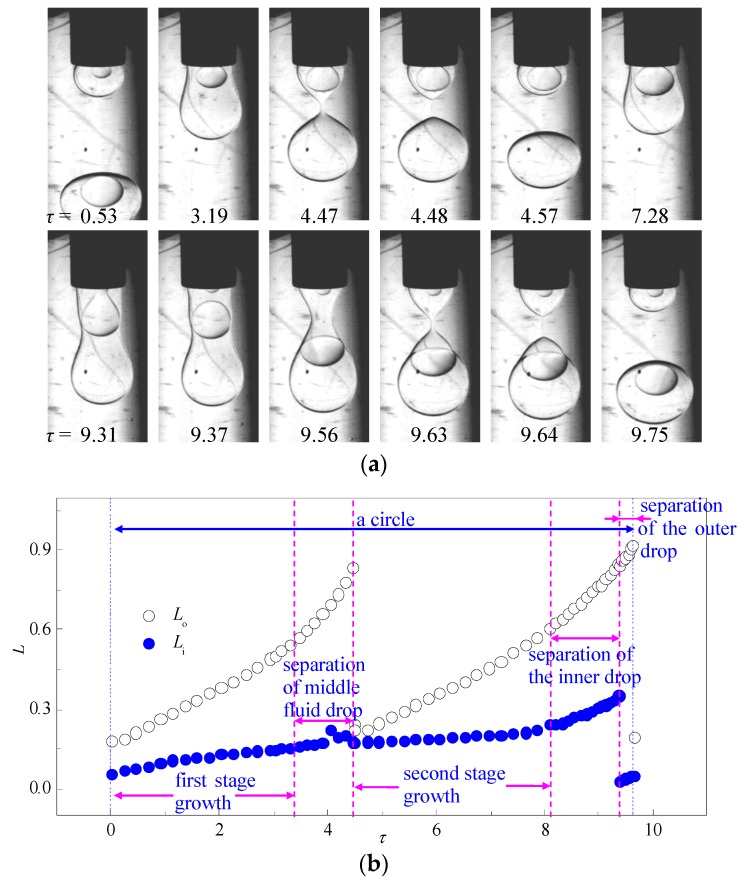
A typical case of single emulsion and double emulsion formed alternately: (**a**) time sequence of interface morphologies; (**b**) variation of the length of the inner and outer drop in one drop formation circle (*q*_i_ = 0.25 mL/h, *q*_m_ = 2.5 mL/h, *q*_o_ = 50 mL/h).

Apart from continuous production of double emulsions, alternative generation of monodispersed single and double emulsion drops is also observed when the flow rate of the middle fluid is much larger than the inner fluid. The drop formation processes are stable and robust with easy control of drop sizes, providing a novel approach for the manufacture of binary materials or dilution of the double emulsions. As shown in Figure 4b, the growing of the inner drop is much slower compared to the outer drop, and the entire drop formation process can be divided as: first stage growth, separation of middle fluid drop, second stage growth, separation of inner drop, and separation of the outer drop. During *τ* = 0–3.19, the faster growing outer drop reaches the critical volume while the inner drop is still growing. A neck on the outer interface develops downstream of the inner drop and the increasing of *L*_o_ is slightly speed up when a single drop of the middle fluid without inner drop is detaching. During this stage, the variation of *L*_i_ fluctuates due to radial squeeze from the outer interface and the inner interface is stretched along the flow direction. After the first pinching-off, both inner and outer interfaces retract back to the tip of the capillaries and the process enters the second growth stage. The axial growth of both inner and outer drops slow down slightly while *L*_o_ is still increasing much faster than *L*_i_. It is worth noticing that the narrowest part of the outer interface is downstream of the inner drop (*τ* = 9.31) when the outer drop begins detaching. However, this part of the interface does not develop into a neck. The pinching-off motion of the inner interface accelerates the fluid flow upstream and the inner drop is pushed through the narrow part of the outer interface. The neck on the outer interface is then developed upstream with the detached inner drop leading to the production of a double emulsion drop.

### 3.4. Influence of the Flow Rates

**Figure 5:**
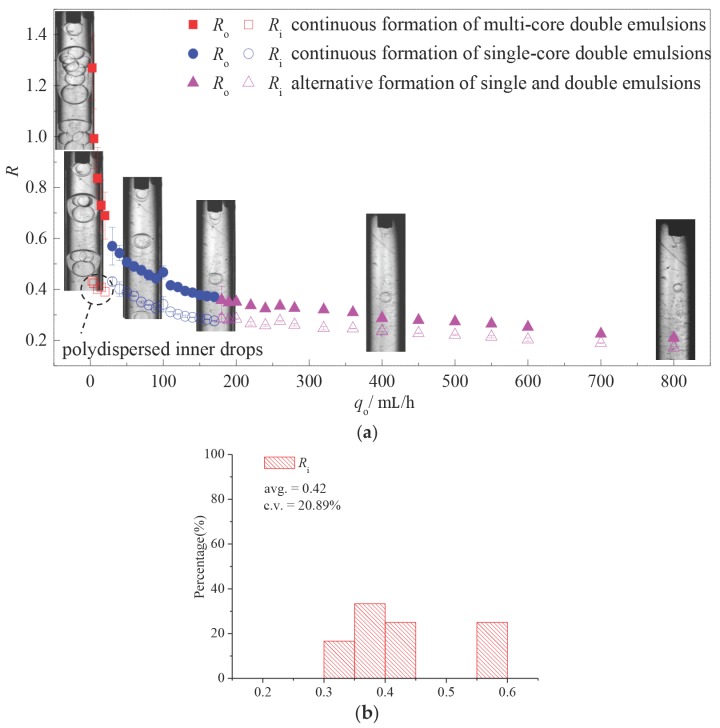
(**a**) Variation of the drop sizes and size distribution via the flow rate of the outer fluid *q*_o_ (*q*_i_ = 2.5 mL/h, *q*_m_ = 2.5 mL/h); (**b**) size distribution of the inner drops of typical multi-core double emulsions (*q*_i_ = 2.5 mL/h, *q*_m_ = 2.5 mL/h, *q*_o_ = 5 mL/h).

Figure 5 plots the variation of non-dimensional radii of both inner drop *R*_i_ and outer drop *R*_o_ with increasing flow rate of the outer fluid *q*_o_. The statistics of size distributions of the drops are noted as the error bars in Figure 5a. The results indicate that, multi-core double emulsions produced by the one-step device are polydispersed in both inner and outer drops and are only generated under the condition when the flow rate of the outer fluid is at the same magnitude with the middle and inner fluid. An example of the size distribution of the inner drops of multi-core double emulsions from a typical case is given in Figure 5b. The size of the inner drops decreases with *q*_o_ slightly due to acceleration of the middle fluid caused by the momentum transfer from the outer fluid. Reduction of the number of inner drops is also observed, leading to drastic decrement on the size of the outer drop *R*_o_. With continuous growth of viscous force, the differences in time required for the inner drops and outer drops to reach critical volumes are lessened, hence generation of monodispersed single-core double emulsions occurs spontaneously. Increasing *q*_o_ results in smaller double emulsion drops, however, the variation of *R*_o_ and *R*_i_ follows the same tendency, which suggested that the increasing viscous force of the outer fluid has no obvious influence on the thickness of double emulsions produced in the one-step co-flowing device. When the flow rate of the outer fluid flow exceeds 180 mL/h, viscous force dominates over interfacial tension that single drops of middle fluid are sheared off between every other double emulsion drop. Under this flow condition, the double emulsions are still highly monodispersed while the single drops are polydispersed due to rupture of the interface repeatedly by the viscous force.

**Figure 6:**
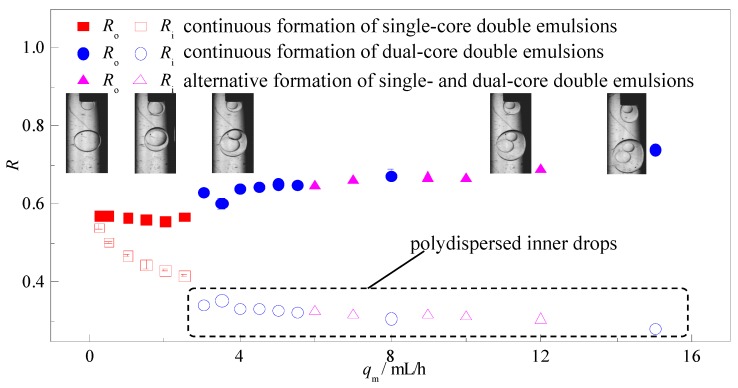
Variation of the drop sizes and size distribution via the flow rate of the middle fluid *q*_m_ (*q*_i_ = 2.5 mL/h, *q*_o_ = 50 mL/h).

Compared to the outer fluid, the flow rate of the middle fluid *q*_m_ has decisive effect on the shell thickness (*R*_o_ − *R*_i_) of the double emulsions and weaker influence on the double emulsion size. Ultra-thin shell double emulsions (see inset in Figure 6) with polydispersity of less than 1% can be produced under low *q*_m_ and the drop size can be actively controlled by *q*_o_. Also, the shell thickness can be adjusted from 5% to 50% of *R*_o_ continuously by increasing *q*_m_ without causing large variation in the size of the double emulsions. A decrease in the inner drop size with *q*_m_ indicates that viscous force from the middle fluid plays critical role in the detaching process of the inner drop and controlling over the shell thickness. However, further increment in *q*_m_ over a threshold alters the number of inner drops and alternative formation of single-core and dual-core double emulsions can be observed. The outer drops are monodispersed while the inner drops are polydispersed due to the same reason in the formation of multi-core double emulsion. Hence, to achieve steady production of monodispersed single-core double emulsions as well maintain better control over the formation process, the flow rate of the middle fluid should be kept at the same magnitude or smaller than the inner fluid.

**Figure 7:**
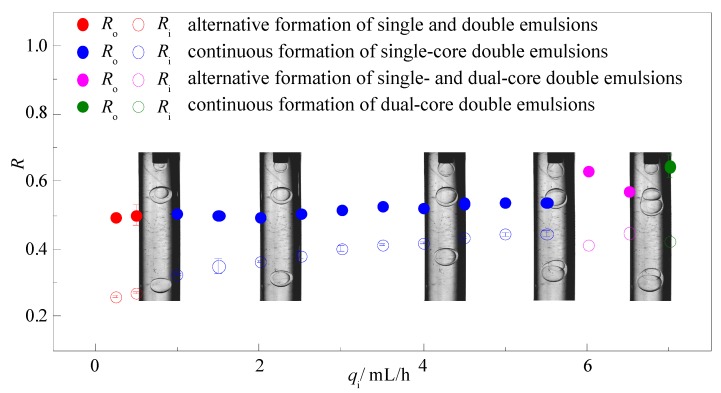
Variation of the drop sizes and size distribution via the flow rate of the inner fluid *q*_i_ (*q*_m_ = 2.5 mL/h, *q*_o_ = 50 mL/h).

Similar to the middle fluid, variation of the flow rate of the inner fluid *q*_i_ has no obvious influence on the drop size of the double emulsions as shown in Figure 7. Under the condition of low *q*_i_, insufficient inner fluid leads to alternative formation of monodispersed single and double emulsions that each take up to 50% percent. As *q*_i_ increases, continuous formation of single core double emulsions can be observed in a wide range that the inner drop size increases slightly with *q*_i_ while the outer drop size remains almost constant. Besides, the shell thickness can only be adjusted in a narrower range by the inner fluid compared to the middle fluid. However, after the increment of *q*_i_ over a threshold, alternative formation of single-core and dual-core double emulsions can be observed with high polydispersity, indicating that low flow rate of the inner fluid is required to obtain steady production of monodispersed double emulsions.

## 4. Conclusions

In this study, a cheap and robust co-flowing microfluidic device is proposed in this paper to generate double emulsions in one step without requirement of surface modification. The growth, deformation, and break-up of the drop interfaces are visualized through high-speed camera to determine the liquid-liquid interaction between the outer and inner drops, in an effort to elucidate the fluid dynamic behaviors during the drop formation processes. The influence of the flow rates of three-phase fluids on flow mode are examined and discussed. The results indicate that the one-step co-flowing microfluidic device is capable of producing the single-core and multi-core O/W/O double emulsions as well as the binary emulsions of single and double emulsions mixture. The detaching of the inner drops accelerates the detaching of the outer drops and hence contributes to drop formation of double emulsions. The manipulation of the flow rates of the outer fluid actively controls the drop formation modes, the number of the inner drops, the drop sizes, as well as the drop size distribution. The drop formation modes are insensitive to the middle and inner fluid flow rates. However, these flow rates significantly affect the shell thickness of the double emulsions.
